# Asthma control and severe exacerbations in patients with moderate or severe asthma in Jilin Province, China: a multicenter cross-sectional survey

**DOI:** 10.1186/s12890-016-0292-3

**Published:** 2016-08-30

**Authors:** Bing-di Yan, Shan-shan Meng, Jin Ren, Zheng Lv, Qing-hua Zhang, Jin-yan Yu, Rong Gao, Chang-min Shi, Chun-feng Wu, Chun-lin Liu, Jie Zhang, Zhong-sen Ma, Jing Liu

**Affiliations:** 1 The Department of Respiratory Medicine, The Second Hospital of Jilin University, Changchun, China; 2 Department of Critical Care Medicine, Zhongda Hospital, School of Medicine, Southeast University, Nanjing, China; 3 The Tumor Centre, The First Hospital of Jilin University, Changchun, China; 4 The Department of Respiratory Medicine, China-Japan Union Hospital of Jilin University, Changchun, China; 5 The Department of Respiratory Medicine, The People’s Hospital of Jilin Province, Changchun, China; 6 The Department of Respiratory Medicine, The 208th Hospital of the Chinese People’s Liberation Army, Changchun, China

**Keywords:** Asthma control, Severe exacerbation, Risk factor, Jilin Province, Asthma medication

## Abstract

**Background:**

No systemic evaluation of asthma control in Jilin Province has been reported. Asthma control might provide the basis for asthma management in this region. A multicenter hospital-based cross-sectional study was performed to investigate the asthma control and related factors for severe asthma exacerbations in patients with moderate or severe asthma in Jilin Province, China.

**Methods:**

The study enrolled 1546 patients in five grade one general hospitals from January to December 2013. Asthma medication, patient self-management, asthma control test (ACT) scores and frequency of severe asthma exacerbations during the follow-up (12 months) were collected via a follow-up questionnaire.

**Results:**

In the study, 889 patients provided a complete follow-up questionnaire. Severe asthma exacerbations occurred in 54.89 % of patients. ACT score ≤15, asthma medication ≤ 3 months, severe asthma, income level lower than average Per Capita Disposable Income (PCDI) and a lower educational level were risk factors of a severe exacerbation.

**Conclusions:**

Poor adherence to asthma medication, poor asthma symptom control, lower income, a low educational level might be possible reasons for the high incidence of severe asthma exacerbations and poor asthma control in Jilin Province of China.

## Background

Asthma is a common disease of airway inflammation with a high prevalence worldwide [1]. Asthma control refers to coming to a steady state after treatment. It has been reported that asthma has not been well-controlled in a large number of asthma patients, as defined by international guidelines [2–4]. A multicenter study of 10 large developed cities in China has reported that 28.7 % and 45.0 % of asthmatic patients achieve full and partial control, respectively, according to the guidelines of the Global Initiative for Asthma (GINA) [5]. However, the data is not sufficient for evaluating the asthma control in China, because China is a large country in which the socioeconomic status, medical insurance coverage, climate and living environments vary considerably between different regions. All of these factors might influence asthma control. Therefore, additional studies on asthma control in different regions of China are needed.

Jilin Province is located in the northeast region of China and characterized by a cold climate, relatively low gross domestic product (GDP) and slow economic development. Until recently, there is few data on asthma control and risk factors for severe asthma exacerbations in Jilin Province. This study aimed to provide useful information in this field. In addition to asthma control test (ACT) questionnaire, we also investigated frequencies of asthma exacerbations. Because there is rare data on risk factors of asthma in China, the study also analyzed the effect of adherence, application of asthma medicine, severity and socioeconomic status on asthma control in Jilin Province so as to further explore the risk factors of asthma in China. This survey will provide useful guidelines for preventing and managing asthma in Jilin Province, China.

## Methods

### Study design and patients

In this multi-center cross-sectional study, 1546 asthmatic patients were enrolled consecutively, who were hospitalized in five general hospitals with grade three in Jilin Province from January 2013 to December 2013.

The patient inclusion criteria were as follow: older than 18 years old; moderate asthma or severe asthma based on the GINA criteria (2012) [6] (that require high intensity treatment to maintain good control or where good control is not achieved despite high intensity treatment); longer than 1 month of diagnosed asthma; longer than 1 year of local residence. Patients with the following diseases were excluded from the survey: severe irreversible organ failure, such as heart, renal and liver failure; severe cerebrovascular disease with concomitant consciousness disturbance; advanced cancer; bronchiectasis; pulmonary embolism; interstitial lung disease; or active tuberculosis. Patients who were unable or unwilling to participate in the survey because of mental or neurological disorders were also excluded. The research protocol was approved by the ethics committee of the Second Hospital of Jilin University. Each patient gave their written informed consent before the survey.

According to the configuration and the ability of the hospital, hospitals in China are classified into grade one hospitals (primary hospitals providing basic health care in communities), grade two hospitals (secondary hospitals affiliated with a medium-size city or district), grade three (comprehensive or general hospitals providing specialist health services, medical education, and scientific studies), and village clinics/community health station [7]. The five hospitals with grade three in the study were the Second Hospital of Jilin University, First Hospital of Jilin University, Jilin Province Hospital, 208th Hospital of the Chinese People’s Liberation Army and China-Japan Union Hospital of Jilin University. These hospitals covered the majority of the geographic regions of Jilin Province. The reason why we enrolled patients with moderate to severe asthma from five hospitals with grade three was that physicians in hospitals with grade three in China have been trained comprehensively about GINA, and could diagnose and educate asthmatic patients correctly, thus the incidence of misdiagnosis and mistreatment would be minimized.

### Survey questionnaire and data collection

Demographic, socioeconomic, and clinical data were collected from hospital medical records. The demographic and socioeconomic data included age, gender, residence, education, occupation, medical insurance type and smoking history. Residence referred to rural or urban residence in China. In Jilin Province, the medical insurance mainly included Jilin provincial insurance (reimbursement ratio ≥80 %), city insurance-civil servants, retirement, employee (reimbursement ratio 70–80 %), city insurance-urban dweller (reimbursement ratio 50–70 %), New Rural Cooperative Medical Insurance (NRCMI, reimbursement ratio 30–50 %) and commercial medical insurance. The clinical data included asthma severity, FEV1 before discharge, hospital duration, atopic status (with clear allergic history or positive specific IgE or positive skin prick test), complications and comorbidities during the hospitalization. The complications and comorbidities consisted of allergic rhinitis, chronic obstructive pulmonary disease (COPD), pneumonia, respiratory failure, circulation system disease (pulmonary hypertension and cor pulmonale, hypertension, coronary heart disease and other heart diseases), nervous system disease, and digestive system disease (e.g., gastro-esophageal reflux disease, gastritis, digestive ulcers). Regular use of ICS (inhaled corticosteroid)/[ICS + LABA (Long-acting beta agonist)]/[ICS+ long-acting muscarinic antagonist (LAMA)] and asthma- associated education from a doctor during hospitalization were also included in clinical data.

Asthma control status and asthma medication during the first 12 months after discharge were collected via a follow-up questionnaire that was completed during each clinical visit (once every 3 months). During the visit, physicians asked the patients the questionnaire and filled in the answers. Review with a physician at least once every three months was defined as regular review or else defined as irregular review. The follow-up questionnaire included questions on several topics. (1) Household income. Because the Per Capita Disposable Income (PCDI) in Jilin Province in 2013 was reported to be 22274.60 Ren min bi (RMB) by the official data from Statistics Department (http://data.stats.gov.cn/), the income levels of the patients were classified into two groups: ≥PCDI group and < PCDI group. Residence referred to rural or urban residence. (2) Self-management of patients included the following information: regularity of a review, and regularity of writing in an asthma diary. (3) Performance of a pulmonary function test (once every three months) and peak flow meter (PFM) utilization. (4) Asthma medication, including the following information: types of medicines, time course of treatment, regularity of application of these medicines, and the reasons for using ICS/(ICS + LABA)/(ICS + LAMA) ≤3 months. (5) ACT scores. In addition to the patient-assessed level of control, ACT questionnaire consisted of four questions concerning symptoms or relief [8]. Based on previous studies [8, 9], the ACT scores were evaluated as follows: ≥20, well-controlled asthma; 16–20, partially controlled asthma; and 5–15, poorly controlled asthma. (6) ACT scores were the mean value of twice testing results, which were completed in the middle and end of follow up year by each patient. A severe asthma exacerbation was defined as hospitalization and emergency department visit because of asthma [10]. The study was conducted in the same way among all institutes.

### Statistical analysis

This study used IBM SPSS Statistics 19.0 software (IBM, Somers, NY, USA) to conduct the statistical analyses. Data were presented as mean ± SD or percentage. A Chi-square test was used to perform a univariate analysis between demographic and clinical factors, and severe asthma exacerbations. A multivariate analysis was conducted using binary logistic regression (Forward: LR), with the significant factors identified from the univariate analysis. Factors were considered significant at *p*-value <0.05. The association was displayed as odds ratios (OR) with 95 % confidence intervals (CIs). A correlation between the ACT score and the frequency of severe exacerbations in asthma patients was tested using a Spearman Correlation Coefficient. Differences with a *p*-value <0.05 were considered significant.

## Results

### Baseline characteristics and clinical date of patients

Collectively, 1546 patients with moderate or severe asthma were enrolled in the study. Their mean age was 55.8 ± 16.8 years old. Baseline characteristics of the patients were shown in Table 1. Approximately 26.9 % of the patients were rural residents, 59.31 % were female patients, 31.82 % possessed middle school and lower education, and 97.67 % had a medical insurance. Former and current smokers were 39.78 % and 9.96 %, respectively.

**Table 1:** General information and demographic data of patients

Characteristic (*n* = 1546)	*N*/%
Age	
≤ 20	67/4.33
21–40	206/13.32
41–60	769/49.74
61–80	434/28.07
> 80	70/4.53
Female gender	917/59.31
Rural area of residence	416/26.91
Education	
Middle school and lower (≤9 years)	492/31.82
High school (9–12years)	669/43.27
University and higher (>12 years)	385/24.90
Occupation	
Jobs in factory or manual labor	198/12.81
Jobs in office (teachers, doctors, civil servants, merchant)	414/26.78
Farmer, poultry or livestock breeder	346/22.38
Retired, housework	481/31.11
Students	54/3.49
Others or unknown	53/3.43
Medical Insurance types	
None	36/2.33
Jilin provincial insurance	132/8.54
City insurance- civil servants, retirement, employee	683/44.18
City insurance- Urban dweller	186/12.03
New Rural Cooperative Medical Insurance (NRCMI)	468/30.27
Commercial medical insurance	41/2.65
Smoking history	
Never smoker	777/50.26
Former smoker	615/39.78
Current smoker	154/9.96

Clinical data of the patients during the hospitalization were summarized in Table 2. The hospital duration was longer (10.15 ± 6.23 days). During the hospitalization, the ratio of the patients received systemic asthma-associated education and treatment with ICS/(ICS + LABA)/(ICS + LAMA) were higher (95.34 %, 92.24 %). Meanwhile, asthma patients got high morbidity rate of complication (81.18 %).

**Table 2:** Clinical characteristics of patients during hospitalization

Characteristic (*n* = 1546)	*N*/%
Severity grades	
Moderate	1157/74.84
Severe	389/25.16
Allergic/atopic status	567/36.67
FEV1 after discharge	
≥ 81 % predicted	53/3.43
50–80 % predicted	755/48.84
≤ 50 % predicted	522/33.76
Not measured	216/13.97
Hospital days	
≤ 7d	712/46.05
8–14d	581/37.58
≥ 15d	253/16.36
Complications and comorbidities	
None	291/18.82
Allergic rhinitis	379/24.51
COPD	452/29.24
Pneumonia	525/33.96
Respiratory failure	248/16.04
Circulation system disease	744/48.12
pulmonary hypertension and cor pulmonale	364/23.54
hypertension	278/17.98
coronary heart disease	325/21.02
other heart diseases	87/5.63
Nervous system disease	160/10.35
Digestive system disease	233/15.07
GORD	132/8.54
Gastritis	129/8.34
Digestive ulcer	106/6.86
Others	125/8.09
Application of ICS + LABA/ICS + LAMA during hospitalization regularly	1426/92.23
Obtaining asthma-associated education from doctor during hospitalization	1474/95.34

*GORD* gastro-oesophageal reflux disease

### Asthma control, asthma medication and severe asthma exacerbations during a 12-month follow-up after discharge

Of the 1546 patients, 889 patients provided a complete follow-up questionnaire. The other 657 patients could not answer all of the questions on the questionnaire because of death (57 cases), personal reasons or unknown causes.

**Fig. 1:**
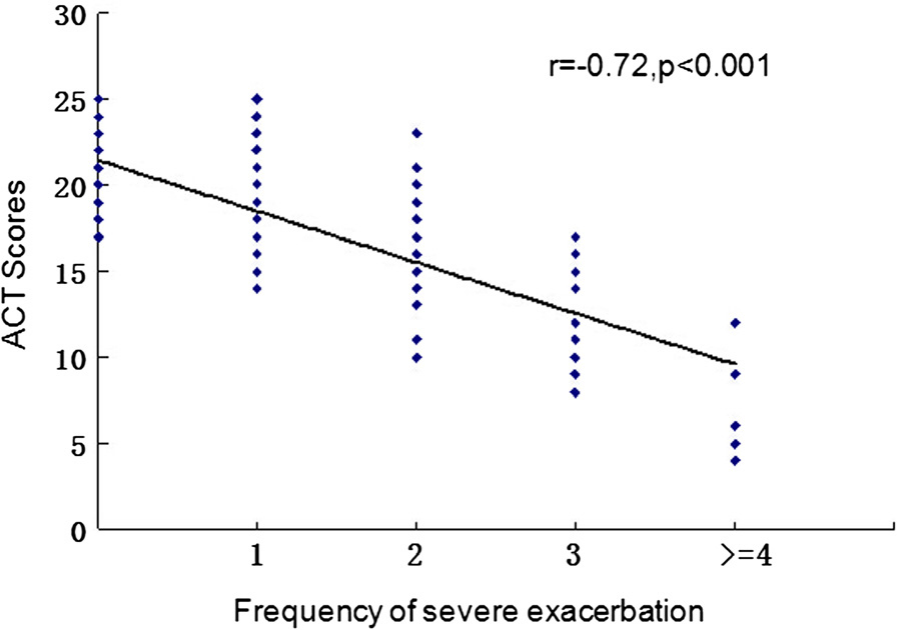
Association of ACT score with frequencies of severe asthma exacerbation

Follow-up data (Table 3) on asthma control, asthma medication and severe asthma exacerbations revealed that 36.33 % of the patients lived with an income below the average PCDI, and only 8.66 % kept in touch with physicians and reviewed regularly. The percentage of patients taking a pulmonary function test, utilizing PFM and writing in an asthma diary regularly were 7.87 %, 3.60 % and 5.29 %, respectively. Only 38.47 % of the patients took ICS/(ICS + LABA)/(ICS + LAMA) regularly for longer than 3 months after discharge regardless of whether they had received asthma education or not. The percentage of patients with an unsatisfactory syndrome control (ACT scores ≤ 20) in Jilin Province was 71.88 %. Severe asthma exacerbations occurred in 54.89 % of the patients, and 11.59 % were admitted to the hospital again within 12 months of discharge. Moreover, ACT scores (it were the mean value of twice testing results, which were completed in the middle and end of follow up year by each patient) were negatively correlated with the frequencies of severe asthma exacerbations (*r* = −0.716, *p* < 0.001, Fig. 1).

**Table 3:** Asthma control, asthma medication and frequency of severe exacerbation in 12 months follow-up after discharge

Characteristic (*n* = 889)	*N*/%
Economic income	
Above the average PCDI	323/36.33
Below the average PCDI	566/63.67
Follow up regularly	77/8.66
Frequency of pulmonary function test	70/7.87
PFM utilization	32/3.60
Writing in an asthma diary regularly	47/5.29
Application of ICS/(ICS + LABA)/(ICS + LAMA) after hospital discharge	
≤ 3 m	547/61.53
3–6 m	218/24.52
> 6 m	124/13.95
Reasons for application of ICS/(ICS + LABA)/(ICS+ LAMA) ≤ 3 m (depend on the answer of patients)	
The medicine was too expensive	391/71.48
Incorrect inhaler technique	55/10.05
Feel symptom was controlled	255/46.62
Others	64/11.70
Application of other asthma medicines	408/45.89
Theophylline	125/14.06
Leukotriene receptor antagonist	204/22.95
OCS	79/8.89
Other drugs (eg. Chinese traditional drugs)	158/17.77
ACT scores (the mean value of one year follow-up)	
≤ 15	281/31.61
16–20	358/40.27
21–25	250/28.12
Frequency of hospital and emergency room admissions in the follow-up	
none	401/45.11
1	173/19.46
2	214/24.07
3	77/8.66
≥ 4	24/2.70
Hospital admission	103/11.59

*RMB* ren min bi, *OCS* oral corticosteroid

### Risk factors for severe asthma exacerbations in the follow-up period

A univariate analysis was performed to explore the risk factors of severe asthma exacerbations (Table 4). The multivariate analysis with a binary logistic regression (Forward: LR) was applied to exclude the mutual interference between the risk factors. Nine risk factors were identified for severe asthma exacerbations in Jilin Province (Table 5). The use of ICS/(ICS + LABA)/(ICS + LAMA) ≤ 3 months was the greatest risk factor for severe asthma exacerbations. The risk factor for poor adherence to treatment was further analyzed. Lower income and education, medical insurance with low reimbursement ratio, female, elderly, and following up irregularly were risk factors for usage of ICS/(ICS + LABA)/(ICS + LAMA) ≤ 3 months (Table 6).

**Table 4:** Univariate analysis of risk factors for severe asthma exacerbation in the follow-up period

Risk factors (n = 889)	*χ*2	*P*-value
Applying of ICS/ICS + LABA/ICS + LAMA ≤ 3 m	152.78	<0.001
ACT scores ≤15	53.33	<0.001
ACT scores ≤20	0.087	0.72
Severe asthma	83.14	<0.001
Following up irregularly	86.32	<0.001
Lower income level than average PCDI	63.43	<0.001
Middle school and lower educational level	58.76	<0.001
Hospital days ≥15d	30.02	0.002
Hospital days >7d	1.55	0.38
Rural residence	22.51	0.005
FEV1 ≤ 50 % predicted	11.13	0.02
FEV1 ≤ 80 % predicted	0.019	0.89
Current and former smoking	2.32	0.18
Female	1.99	0.24
Older > 60 years old	11.11	0.02
Older > 40 years old	0.07	0.88
New Rural Cooperative Medical Insurance (NRCMI)	7.53	0.05
Jobs in factory or manual labor	1.86	0.36
Jobs in office	1.67	0.34
Farmer, poultry or livestock breeder	6.19	0.06
Retired, housework	0.57	0.33
Former smoke	0.18	0.64
High school	0.05	0.77
Complications and comorbids (n/%)		
Allergic status	5.07	0.08
Allergic rhinitis	2.05	0.14
pneumonia	0.18	0.65
Circulation system disease	0.87	0.16
Nervous system disease	0.17	0.69
Digestive system disease	1.79	0.30
Application of other asthma medicines (n/%)	0.06	0.71

The Chi-squared test was used to perform univariate analysis between the demographic and clinical factors and severe exacerbation of asthma

**Table 5:** Multivariate analysis of risk factors for severe asthma exacerbations in the follow-up period

Risk factors (n = 889)	OR	CI	*P*-value
Applying of ICS/(ICS + LABA)/(ICS + LAMA) ≤ 3 m	5.81	3.23-10.47	<0.001
ACT scores ≤15 (mean value of one year follow-up)	4.49	2.28-8.89	<0.001
Severe asthma	4.18	2.54-8.53	<0.001
Lower income level than average PCDI	2.67	1.44-4.95	0.002
Middle school and lower educational level	2.43	1.38-4.29	0.002
Hospital days ≥15d	2.41	1.44-4.04	<0.001
Rural residence	2.15	1.13-4.10	0.02
FEV1 ≤ 50 % predicted before discharge	1.86	1.16-3.0	0.01

A multivariate analysis was conducted using binary logistic regression (Forward: LR), with the significant factors identified from the univariate analysis. The association was displayed as odds ratios (OR) with 95 % confidence intervals (CIs)

**Table 6:** Multivariate analysis of risk factors for application of ICS + LABA/LAMA ≤ 3 months in the follow-up period

Risk factors (n = 889)	OR	CI	*P*-value
Middle school and lower education level	3.96	1.98-7.91	<0.001
Lower income level than average PCDI	2.51	1.25-5.01	<0.001
New Rural Cooperative Medical Insurance (NRCMI)	2.49	1.13-5.94	0.024
Female	2.33	1.39-3.89	0.001
Older > 60 years old	2.3	1.31-4.01	0.004
Following up irregularly	2.11	1.23-4.59	0.01

A multivariate analysis was conducted using binary logistic regression (Forward: LR), with the significant factors identified from the univariate analysis. The association was displayed as odds ratios (OR) with 95 % confidence intervals (CIs)

## Discussion

It was the first multicenter hospital-based cross-sectional study to comprehensively investigate status of asthma control and risk factors for severe asthma exacerbations in patients with moderate or severe asthma in Jilin Province in northeast China. Based on the ACT scores, the current study found good asthma control in 31.61 % of the patients with moderate or severe asthma; partial asthma control in 40.27 %; and poor asthma control in 28.12 %. The percentage of the patients with severe asthma exacerbations during the follow-up period was 54.89 %. These findings suggest unsatisfactory asthma control. Moreover, the study found that the ACT scores were negatively correlated with the frequency of severe asthma exacerbations during the follow-up period, and ACT scores ≤ 15 was identified as an important risk factor for severe asthma exacerbations. The finding was in accordance with the data presented by Haselkorn T [11]. The differences between ACT scores and frequency of severe asthma exacerbations were that ACT scores were often required from the patient's subjective feeling, and revealed four recent weeks of asthma control rather than one year.

Poor adherence was shown in our study. Only 38.4 % of the patients continued their medication for more than 3 months after discharge, and only 8.66 % of the patients were reviewed regularly by physicians, which is lower than the data found in a study conducted in USA [12]. The study also showed that within one year after discharge, severe asthma, rural and poor patients tended to have lower ACT scores and higher frequencies of severe asthma exacerbations.

Asthma control assessments contain symptom control assessment and risk factor assessment. It has been reported that overestimating asthma control based on ACT scores will result in inadequate treatment or even termination of medication [13–15]. Thus, we not only studied ACT scores, but also investigated the frequency and risk factors of severe asthma exacerbations in the patients in the current study. The use of ICS/(ICS + LABA)/(ICS + LAMA) ≤ 3 months was found to be the greatest risk factor for severe asthma exacerbations, which was in line with previous findings [16–19]. In addition to ICS/(ICS + LABA)/(ICS + LAMA) ≤ 3 months, the study found that ACT scores ≤15, asthma severity, a junior high school and lower educational level, an income level lower than PCDI, hospital days ≥15 days, rural residence and FEV1 ≤ 50 % predicted were also risk factors for severe asthma exacerbations. Previous studies have reported that FEV1 < 60 % predicted is a risk factor for exacerbations [20, 21]. However, the study found that FEV1 < 50 % predicted was a more significant criterion than FEV1 < 60 % predicted for severe asthma exacerbations. This difference might be attributed to the fact that the pulmonary function testing has primarily been conducted in convalescence, immediately following an acute exacerbation, when pulmonary function is not totally recovered. In the study, 71.48 % of the patients who did not take the ICS/(ICS + LABA)/(ICS + LAMA) for more than 3 months complained that the medicines were too expensive (300–600 RMB per month). Our study revealed that lower income and education, and medical insurance with low reimbursement ratio were main risk factors for poor adherence of asthma treatment, and 71.48 % of patients stopped the therapy of ICS/(ICS + LABA)/(ICS + LAMA) ahead of schedule, due to expensive price of medicines. So reduced out-of-pocket expenses [22] or free drugs may improve medication adherence from the government level. Otherwise, 45.89 % of asthma patients used other drugs to instead of ICS/(ICS + LABA)/(ICS + LAMA), for example theophylline, which has been recently shown to have antiinflammatory effects in asthma at lower concentrations, is used commonly in China for cheaper price [23]. Meanwhile, some traditional Chinese medicine had been used irregularly. So a multi-center, cross-sectional study about certain cheaper drugs for asthma control to improve adherence to self-administered medications should be designed well in further.

These findings suggested that lower socioeconomic and educational levels and limited reimbursement from insurance were the primary obstacles to regularly using their asthma medicine. These were in accordance with previous data showing that socioeconomic problems are major risk factors for asthma-related deaths [24]. One explanation is that government revenue, education and medical expenditures of Jilin Province are relatively low in China [6]. Furthermore, a low household income and low educational level were shown to not only decrease the adherence of asthma medication but also independently increase the risk of severe asthma exacerbations in the present study. Similarly, it has been shown that the risk of severe life threatening asthma (SLTA) increases with the lack of formal income [25]. This indicated that higher household income, increased education and medical expenditures, and higher reimbursement from insurance might aid in prevention of severe asthma exacerbations and contribute to asthma control improvement.

Atopic status and infection are both important factors for acute attack of asthma. We found 36.67 % of patients had atopic status in our study, but atopic status of patients was not risk factor for severe asthma exacerbations in the follow-up period. The comorbidity of pneumonia, which reflected partly infection in lung, was not associated with severe asthma exacerbations in the follow-up period.

Smoking has been reported to be a risk factor for poorly controlled asthma and an impaired corticosteroid response [26]. In contrast, smoking was not a significant risk factor for severe asthma exacerbations in our survey, which might be due to the lower percentage (9.96 %) of asthma patients who were currently smoking compared with other studies. The percentage of former smoker was 39.78 %. This suggested that many patients might quit smoking after diagnosis of asthma. Another reason might be difference patient resources.

The study has some limitations. There may be a selection bias because the asthma patients were enrolled only from five first-class hospitals in Jilin Province. Another limitation is the high dropout rate that 889 of 1546 patients provided a complete follow-up questionnaire. Furthermore, it is difficult to evaluate the effect of season and airway infection on severe asthma exacerbation each time, because we collected the data of severe asthma exacerbations of patients in follow up visit per three months retrospectively, but not immediately when the acute exacerbations happened. And some information in detail was not complete because the hospitalization and emergency department visit of patients were not in original hospitals sometimes. Otherwise, it is difficult to evaluate the infection situation each time, due to the partly abuse of antibiotics in China. Thus, increasing the frequency of follow-up visits and establishing the network of hospitals with different grades would be solutions. Finally, follow-up studies after discharge might increase the understanding and condition of asthma control. Further large-scale studies are warranted to validate the findings of this study.

## Conclusion

Collectively, this study found unsatisfactory asthma control and a high frequency of severe asthma exacerbation in Jilin Province, which were mainly due to poor adherence to asthma medication, poor asthma symptom control, low income, a low educational level and a low proportion of reimbursements from insurance companies. Our results provide basic data for improving asthma control in Jilin Province and will help physicians instruct patients on how to avoid the risk factors associated with asthma exacerbation.

## Abbreviations

ACT: Asthma control test
CI: Confidence interval
COPD: Chronic obstructive pulmonary disease
FEV1: Forced expiratory volume in one second
GDP: Gross domestic product
GINA: Global Initiative for Asthma
ICS: Inhaled corticosteroids
LABA: Long acting beta 2 agonist
LAMA: Long-acting muscarinic receptor antagonists
NRCMI: New rural cooperative medical insurance
OR: Odds ratio
PCDI: Per capita disposable income
PEF: Peak expiratory flow
PFM: Peak flow meter
SABA: Short acting beta 2 agonist
SLTA: Severe life threatening asthma
